# Single center experience of the impact of artificial intelligence image analysis software on short-term prognosis of non-small cell lung cancer

**DOI:** 10.3389/fonc.2025.1633035

**Published:** 2025-11-26

**Authors:** Luyuan Chang, Siyu Dong, Ailijiang Kadeer, Shilong Song, Tingting Guo, Fei Liu

**Affiliations:** 1 The First Department of Clinical Medicine, Bengbu Medical University, Bengbu, Anhui, China; 2 Department of Oncology, First Affiliated Hospital of Bengbu Medical University, Bengbu, Anhui, China; 3 Department of Radiotherapy, First Affiliated Hospital of Bengbu Medical University, Bengbu, Anhui, China; 4 Department of Surgical Oncology, The Second Affiliated Hospital of Bengbu Medical University, Bengbu, Anhui, China

**Keywords:** artificial intelligence, non-small cell lung cancer, prognosis, imaging features, computer-aided diagnosis

## Abstract

**Objective:**

To explore the single center experience of the value of artificial intelligence image analysis software in short-term prognostic assessment of non-small cell lung cancer.

**Methods:**

Artificial intelligence image analysis software was used to analyze typical cases of NSCLC in our hospital; 450 patients diagnosed with NSCLC were selected as research subjects, Single-factor and multi-factor COX proportional hazards regression were used to analyze the imaging features that affect the short-term survival prognosis (progression/death within 12 months) of NSCLC patients, and the short-term prognostic predictive value of each independent predictor factor was analyzed through the receiver operating characteristic (ROC) curve.

**Results:**

The artificial intelligence image analysis software can accurately identify and segment tumor areas, extract key features such as tumor size, shape, and texture, and help doctors diagnose and treat patients more efficiently and accurately. COX regression analysis showed that the maximum diameter of the tumor, spiculation sign, vascular bundle sign, pleural indentation sign, calcification and lymph node metastasis are all imaging features that affect the prognosis of NSCLC patients. The ROC curve shows that the areas under the curve (AUC) of the six factors are 0.676, 0.768, 0.689, 0.696, 0.713, 0.810, respectively, with 95% confidence intervals (95%CI) are =0.576~0.740, 0.663~0.847, 0.610~0.763, 0.590~0.781, 0.614~0.808, 0.716~0.886 respectively. The precision-recall curve of lymph node metastasis and spiculation sign performed best. Even under high recall rate, the precision rate remained above 0.7. The model quality score showed that lymph node metastasis had the highest score (0.74) and spiculation sign was 0.66.

**Conclusion:**

The imaging analysis software based on artificial intelligence can significantly improve the accuracy of assessment of NSCLC patients, help improve the short-term prognosis of patients, and has short-term clinical application value.

## 1 Introduction

Non-small cell lung cancer (NSCLC) is the main subtype of lung cancer, accounting for approximately 85% of all lung cancer cases. It is mainly divided into adenocarcinoma, squamous cell carcinoma and large cell carcinoma based on histological characteristics (1, 2). The early clinical manifestations of NSCLC include cough, hemoptysis, chest pain, shortness of breath, etc. As the disease progresses, it may cause symptoms such as pleural effusion, dysphagia, and hoarseness (3, 4). The harm of NSCLC is not limited to the lungs. Late-stage lesions often metastasize to the brain, liver, bones and other organs, causing systemic symptoms and seriously threatening the patient’s life (5, 6). In view of the complex biological characteristics and prognostic differences of NSCLC, accurate imaging analysis and early intervention for patients have become the key to improving survival rate and quality of life (7, 8). Traditional image analysis methods have certain limitations, are highly subjective, and are difficult to capture potential prognostic information of tumors, making it often difficult to achieve accurate diagnosis (9). Aerts et al. (36) proposed a quantitative radiomics framework for the first time in Nature Communications in 2014, laying the foundation for non-invasive assessment of NSCLC. On this basis, artificial intelligence image analysis software has been widely used in the diagnosis and treatment monitoring of various diseases such as lung cancer, breast cancer, and stroke (10). Hosny et al. (37) confirmed in “PLoSMedicine” in 2018 that the 3DCNN model can accurately predict the prognosis of NSCLC through CT images. Wang et al. (11) found that the artificial intelligence algorithm performed well in identifying tumor areas in pathological images through deep learning models, and could distinguish subtle differences in cell and tissue structure, which was helpful for tumor grading and metastasis detection. Sheth et al. (12) have shown that artificial intelligence analysis can greatly improve the diagnostic accuracy of breast cancer by integrating image data with pathology, genes and clinical features. Davatzikos et al. (13) found that the artificial intelligence toolkit can extract quantitative image features such as tissue texture, tumor volume and morphology from multiple imaging modalities through advanced image processing and machine learning algorithms, which can help doctors achieve accurate tumor location and feature analysis. Therefore, artificial intelligence image analysis technology uses algorithms such as machine learning and deep learning to automatically analyze and quantify a large amount of imaging data, which can more sensitively identify imaging abnormalities, detect early microscopic lesions, avoid manual errors, and achieve more accurate staging and therapeutic efficacy evaluation of the disease (14, 15). Based on this, this study will use artificial intelligence image analysis software to conduct in-depth analysis of typical NSCLC cases and explore the imaging characteristics that affect the prognosis of NSCLC patients, with a view to providing important reference value for medical decision-makers. Based on this, this study will use artificial intelligence image analysis software to conduct in-depth analysis of typical cases of NSCLC, and explore the imaging characteristics that affect the short-term survival prognosis (progression, death) of NSCLC patients within 12 months through COX proportional hazard regression, in order to provide important reference value for short-term clinical evaluation for medical decision-makers.

## 2 Materials and methods

### 2.1 General information

This study is a single-center retrospective observational study, and the study time is from August 2022 to August 2024. The case data and chest CT images of NSCLC patients who meet the standards were retrospectively collected through the hospital’s electronic medical record system and PACS imaging platform, and clinical prognosis outcomes were evaluated based on follow-up information. All data were treated anonymously, and the research protocol was approved by the hospital ethics committee. 450 NSCLC patients treated in our hospital from August 2022 to August 2024 were selected as the research subjects. Inclusion criteria: (1) NSCLC confirmed by histopathology or cytology; (2) age ≥18 years old; (3) complete and clear imaging data, including at least chest CT or PET-CT images; (4) complete clinical data. Exclusion criteria: (1) Combined with other types of malignant tumors; (2) Combined with severe cardiovascular disease, renal failure and chronic liver disease; (3) Pregnant or lactating women; (4) The size of the lesion cannot be accurately measured in imaging evaluation or the location of the lesion is not suitable for AI software analysis; (5) Combined with severe mental illness or cognitive dysfunction. In order to reduce selection bias, this study only included NSCLC patients who were first diagnosed and treated for the first time, excluding those with postoperative recurrence, multiple primary cancers, or those who had been evaluated before receiving targeted/immunotherapy intervention; all cases were confirmed by imaging and pathology, and completed at least 12 months of follow-up (the length of follow-up was based on short-term prognostic evaluation goals, aiming to capture disease progression events within 12 months). Follow-up methods include regular outpatient review, electronic medical record tracking and telephone follow-up to ensure the completeness of prognostic event records.

### 2.2 CT examination method

Chest scans were performed using American GERevolution spiral CT and United Imaging uCT760 spiral CT scanners. The patient lies supine on the examination bed, raises his arms above his head, inhales deeply and holds his breath to complete the scan. The scan range is from the apex of the lungs to the top of the diaphragm.

Scanning parameters: (1) Revolution: tube voltage is 100-120kVp, tube current is 200-300mA, pitch is 0.9-1.2, scanning speed is 0.5s/revolution, matrix 512×512, FOV32.0cm, reconstruction algorithm: Stand algorithm, thin layer reconstruction layer thickness is 1.25mm. (2) uCT760: Tube voltage is 100-120kV, tube current is 150-250mA, pitch is 1.0-1.2, scanning speed is 0.35s/turn, matrix 512×512, FOV32.0cm, reconstruction algorithm: B_SOFT_B, thin-layer reconstruction layer thickness is 1.5mm. Window settings: mediastinal window (window level 40HU, window width 350HU); lung window (window level -600HU, window width 1200HU).

### 2.3 CT imaging feature extraction

#### 2.3.1 Overview of artificial intelligence analysis software and functions

The artificial intelligence image analysis software used in this study was developed based on the U-Net architecture in convolutional neural networks (CNNs) (this architecture has accurate boundary recognition in medical image segmentation and is suitable for fine segmentation of lung tumors). The model is run on 3D chest CT data (it can completely capture the three-dimensional shape, volume and relationship between the tumor and the surrounding tissue, which is better than 2D data). It has the ability to automatically segment lung nodules, extract radiomic features and quantitative analysis. The system was developed by Shanghai Yitu Medical Technology Co., Ltd., and the training data set is constructed using a combination of “hospital historical data + public data sets”: the hospital data are chest CT images of 1,200 NSCLC patients pathologically diagnosed in our hospital from January 2018 to July 2022 (ethical approval and anonymization), and the public data comes from a subset of 800 NSCLC images from the TCIA database (in compliance with the open protocol); the two types of data have been preprocessed (normalization, noise reduction, enhancement) The random stratified sampling method was then used to divide the data into a training set (1400 cases), a validation set (200 cases), and an internal independent test set (400 cases) in a ratio of 7:1:2. The stratification basis was tumor pathological subtype (adenocarcinoma/squamous cell carcinoma) and TNM stage (I- Phase II/Phase III-IV), ensuring that the distribution of cases in each data set is consistent with the overall data to avoid sampling bias; during the training process, the validation set is used to adjust the model hyperparameters (such as learning rate, number of iterations) in real time, and the internal independent test set is used to initially evaluate the basic performance of the model. In order to further verify the generalization ability of the model, this study conducted additional external validation: using the chest CT images of 300 NSCLC patients in the AAPM (American Association of Physicists in Medicine) lung cancer imaging database who did not participate in model training (all pathologically confirmed, image layer thickness ≤1.5mm, in line with the analysis standards, and authorized for use by the database) as an external independent validation set to evaluate the segmentation stability of the model in non-hospital and non-TCIA data sets. Taking the tumor area manually segmented by 2 radiologists with more than 5 years of experience in chest imaging diagnosis (independent of the collaborating physicians in Section 1.3.2) as the “gold standard”, the model segmentation performance index was calculated: in the internal independent test set, the Dice similarity coefficient (DSC) of tumor area segmentation was 0.89±0.04, and the sensitivity was 92.3% (95%CI: 89.1%-94.8%), the specificity was 94.1% (95%CI: 91.5%-96.2%); in the external validation set, the DSC was 0.86±0.05, the sensitivity was 89.7% (95%CI: 85.3%-92.8%), and the specificity was 92.5% (95%CI: 89.0%-94.9%), indicating that the model segmentation accuracy and generalization met the requirements for clinical application. The main modules include image preprocessing, automatic identification and annotation of tumor areas, extraction of key imaging parameters (maximum tumor diameter, texture analysis, etc.) and risk stratification prediction. The operation process is: input the original CTDICOM file, the system generates a structured report and annotated images within 3 minutes, and the extraction parameters can be exported to Excel or SPSS for statistical analysis.

#### 2.3.2 Collaboration process between AI and radiologists

In order to ensure the accuracy and consistency of image feature extraction, all chest CT images are first automatically segmented and analyzed by AI software, and the output includes quantitative or structural features such as the maximum diameter, volume, density, texture, and boundary shape of the tumor. Subsequently, two radiologists with more than three years of experience, under the condition of complete blindness to the patient’s clinical outcomes (such as prognostic grouping results), pathological diagnosis conclusions and medical history information, first independently completed the review and manual fine-tuning of the AI ​​results, as well as the interpretation of features that are difficult to identify or highly subjective in AI (such as pleural depression sign, cavitation sign, pleural traction, etc.); after both of them completed independent reviews, the results were cross-compared. When there is inconsistency between the AI ​​output and the manual interpretation results, or there are differences in the independent interpretation conclusions of two doctors, the two doctors will jointly read the films and reach an agreement based on clinical imaging diagnosis standards, and finally unify the feature data for statistical analysis. In addition, this study used the Cohen’skappa coefficient to evaluate the inter-observer consistency of the independent interpretation results of two doctors. The calculated kappa value was 0.83 (95% CI: 0.77-0.89), indicating that the consistency of the two interpretations reached an “excellent” level. This collaborative process implements the “AI pre-screening + blind independent review by doctors + two-person consensus + consistency verification” mechanism to improve the accuracy, objectivity and reliability of feature recognition.

#### 2.3.3 Image feature definition and classification description

The chest CT images of NSCLC patients included in this study were analyzed for imaging characteristics, including: maximum tumor diameter, tumor location, clear edge, lobulation sign, spiculation sign, cavitation sign, blood vessel clustering sign, air bronchus sign, bronchial truncation sign, pleural dent sign, chest wall invasion, pleural traction, pleural effusion, satellite lesions, calcification, intrapulmonary metastasis, lymph node metastasis and other characteristics. When the two doctors disagreed on the CT imaging signs of tumors, they negotiated to reach a consensus. (1) Maximum tumor diameter: the maximum diameter of the tumor on the cross-section; (2) Tumor location: Tumors originating from the bronchi of the lung segment and above are classified as central lung cancer, and tumors originating from the bronchi below the lung segment are classified as peripheral lung cancer; (3) Tumor edge: divided into clear edge and blurred edge; (4) Lobulation sign: refers to the outline of the lesion showing multiple arc-shaped convexities, and the arcs are concave to form a lobular shape. (5) Spicule sign: refers to the radial thin short linear density increase around the lesion. (6) Cavitation sign: refers to the appearance of irregular low-density areas or lacunae within the tumor; (7) Vascular bundle sign: refers to one or several small pulmonary blood vessels being pulled toward the lesion, gathering and shifting, and interrupting or penetrating the lesion; (8) Air bronchus sign: refers to the presence of air-containing bronchial branch shadows in the lesion area; (9) Bronchial cross section Broken sign: refers to the bronchus being blocked by the lesion and the distal bronchus disappears; (10) Pleural depression sign: refers to the depression at the edge of the tumor and pulling it to the pleura, showing that one side of the tumor is connected to the pleura and forming a local depression; (11) Chest wall invasion: refers to the fusion of the tumor boundary with the chest wall tissue, and the edge is unclear, and the tumor tissue moves toward the chest wall muscles, ribs, etc. Structural penetration leads to interruption of pleural continuity; (12) Pleural traction: refers to the slight bending of the pleura in the direction of the tumor but does not destroy the integrity of the pleura; (13) Pleural effusion: refers to the observation of free or wrapped fluid density shadows in the pleural cavity; (14) Satellite lesions: refers to small nodules that appear around the primary tumor, with a shape similar to the main tumor and a solid appearance (15) Calcification: refers to small bright white spots, plaques or larger high-density areas within the tumor; (16) Intrapulmonary metastasis: refers to the occurrence of isolated cancer nodules in the ipsilateral, contralateral or bilateral lung lobes; (17) Lymph node metastasis: refers to lymph node enlargement around the tumor or in specific lymphatic drainage areas.

### 2.4 Statistical analysis

Data were analyzed using SPSS27.0 statistical software. Continuous variables were tested for normality by the Shapiro-Wilk test. Those that conformed to the normal distribution were expressed as mean ± standard deviation (±s). Comparisons between groups were performed using independent samples t-test. Enumeration data (such as clear tumor edges, calcification, lymph node metastasis, etc.) were expressed as frequency (n) and percentage (%). Differences between groups were expressed using χ² test. All tests were two-tailed tests. In the data preprocessing stage, since the total missing rate of clinical and imaging features was <3%, continuous missing data (such as the maximum diameter of the tumor) were filled using the multiple imputation method, and missing data of categorical variables (such as pleural traction sign) were filled using the nearest neighbor matching method. The variables are clearly coded: the COX regression dependent variable is the binary variable “survival time-outcome status” (time: the time from diagnosis to the outcome, unit: months; outcome: 1=progression/death within 12 months, 0=no progression and survival within 12 months (for those without outcome events, the censoring time will be 12 months of follow-up); the maximum diameter of the continuous variable tumor is substituted according to the actual measured value (unit: cm); the binary independent variables (such as spiculation sign, vascular bundle sign, calcification, etc.) are all coded with “1=yes, 0=no”. Before multivariate COX proportional hazards regression analysis, the proportional hazards assumption was tested through Schoenfeld residuals (all variables P>0.05, meeting the proportional hazards assumption), and multicollinearity was tested through the variance inflation factor (VIF) (VIF value 1.0 2~1.35, no significant collinearity); variables with statistically significant differences in univariate COX regression analysis were selected and included in the multifactor model, HR values (hazard ratios) and 95%CI were calculated to clarify their independent impact on short-term survival prognosis (progression/death within 12 months). The predictive efficacy of each factor was evaluated through the ROC curve and AUC value (the closer the AUC is to 1, the stronger the predictive ability), and P<0.05 was considered a statistically significant difference.

## 3 Results

### 3.1 Artificial intelligence image analysis

**Figure 1:**
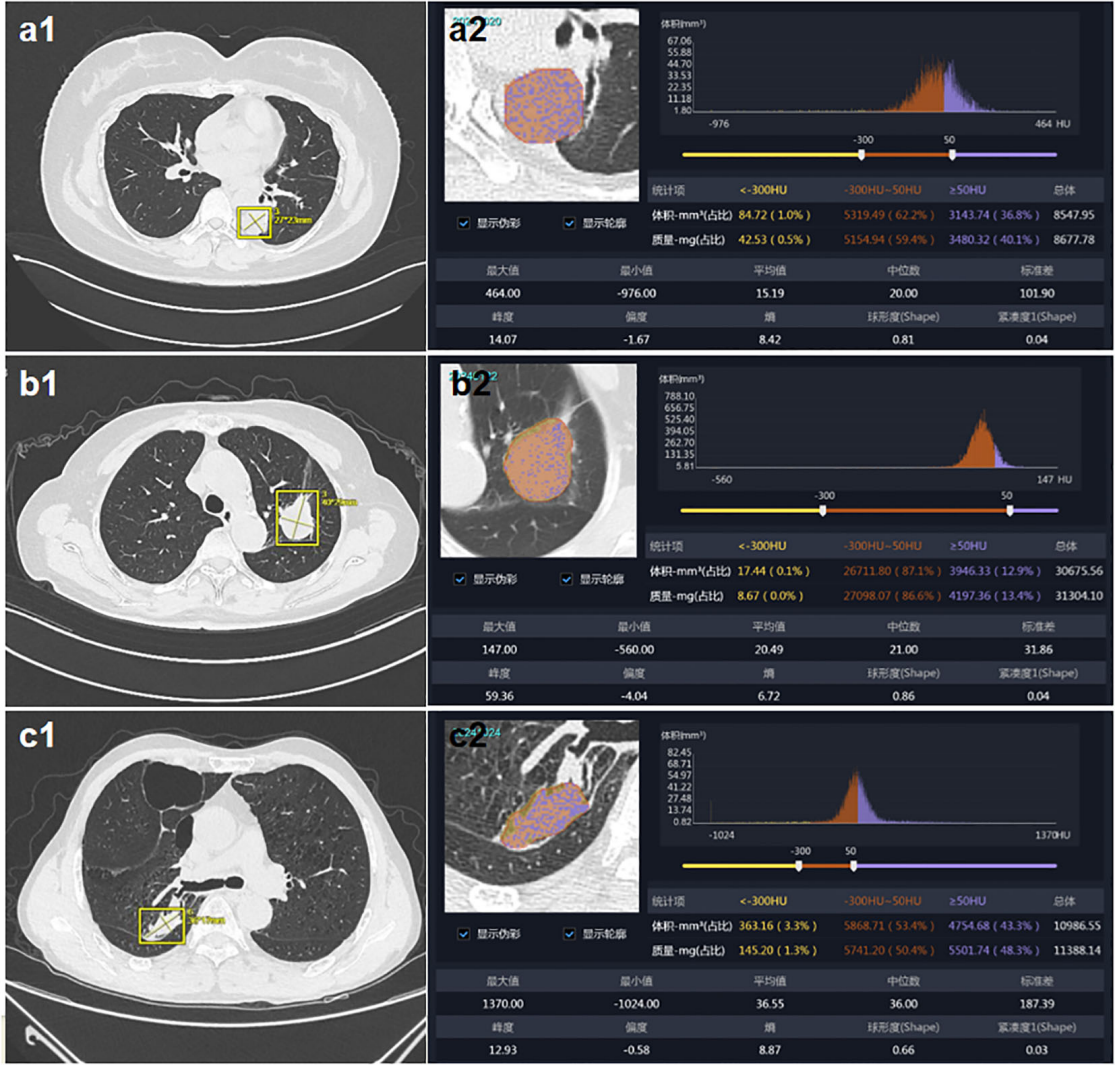
CT imaging features based on artificial intelligence analysis. **(a)** patient 1: female, age 40, tumor size is ≥3mm, with tumor dimensions of 27×23mm. **(b)** patient 2: female, age 68, tumor size is ≥3mm, with tumor dimensions of 40×29mm. **(c)** patient 3: male, age 66, tumor size is ≥3mm, with tumor dimensions of 36×17mm.

Based on artificial intelligence image analysis, CT quantitative parameters are obtained and the average diameter of the patient’s pulmonary nodules is calculated. It can be seen that the artificial intelligence image analysis software can quickly locate the suspicious lesion area, accurately segment the tumor boundary, automatically identify quantitative parameters such as tumor size, location, and texture characteristics, and provide quantitative analysis results. A typical case is shown in **Figure 1**.

### 3.2 Baseline information and CT imaging characteristics of study subjects

A total of 450 research subjects were included in this study, and the core characteristics are as follows: Baseline data: 72.22% (325 cases) were male, 64.00% had a history of smoking; 38.67% and 30.44% had hypertension and diabetes respectively. Pathology was dominated by adenocarcinoma (63.56%), TNM stage III-IV accounted for 66.00%; tumors with maximum diameter ≥4.5cm accounted for 67.78%, and peripheral type was more common (59.78%). CT imaging features: 76.67% had clear tumor margins, and rare signs such as lobulation sign, spiculation sign, and vascular bundle sign accounted for less than 20%; air bronchus sign (71.33%), pleural effusion (66.89%), and pleural traction (60.67%) were common signs. Among the metastasis-related features, lymph node metastasis accounted for 59.78% and intrapulmonary metastasis accounted for 29.33%. See **Table 1**.

**Table 1:** Baseline data and CT imaging characteristics of the study subjects.

Item		Total
Age (years, $\bar{\text{x}}$ ± s)	≥60	
	<60	
Gender [n (%)]	Male	325(72.22)
	Female	125(27.78)
Smoking History [n (%)]	Yes	288(64.00)
	No	162(36.00)
Hypertension History [n (%)]	Yes	174(38.67)
	No	276(61.33)
Diabetes History [n (%)]	Yes	137(30.44)
	No	313(69.56)
Histopathological Type [n (%)]	Squamous Cell Carcinoma	164(36.44)
	Adenocarcinoma	286(63.56)
TNM Stage [n (%)]	Stage I–II	153(34.00)
	Stage III–IV	297(66.00)
Maximum Tumor Diameter (cm, $\bar{\text{x}}$ ± s)	≥4.5	305
	<4.5	145
Tumor Location [n (%)]	Central	181(40.22)
	Peripheral	269(59.78)
Well-Defined Margin [n (%)]	No	105(23.33)
	Yes	345(76.67)
Lobulation Sign [n (%)]	No	401(89.11)
	Yes	49(10.89)
Spiculation Sign [n (%)]	No	371(82.44)
	Yes	79(17.56)
Cavitation Sign [n (%)]	No	233(51.78)
	Yes	217(48.22)
Vascular Convergence Sign [n (%)]	No	396(88.00)
	Yes	54(12.00)
Air Bronchogram Sign [n (%)]	No	129(28.67)
	Yes	321(71.33)
Bronchial Cutoff Sign [n (%)]	No	344(76.44)
	Yes	106(23.56)
Pleural Indentation Sign [n (%)]	No	270(60.00)
	Yes	180(40.00)
Chest Wall Invasion [n (%)]	No	262(58.22)
	Yes	188(41.78)
Pleural Traction [n (%)]	No	177(39.33)
	Yes	273(60.67)
Pleural Effusion [n (%)]	No	149(33.11)
	Yes	301(66.89)
Satellite Nodules [n (%)]	No	269(59.78)
	Yes	181(40.22)
Calcification [n (%)]	No	334(74.22)
	Yes	116(25.78)
Intrapulmonary Metastasis [n (%)]	No	318(70.67)
	Yes	132(29.33)
Lymph Node Metastasis [n (%)]	Yes	269(59.78)
	No	181(40.22)

*indicates P < 0.05.

### 3.3 COX single-factor and multi-factor regression analysis of factors influencing short-term prognosis of patients with NSCLC

**Figure 2:**
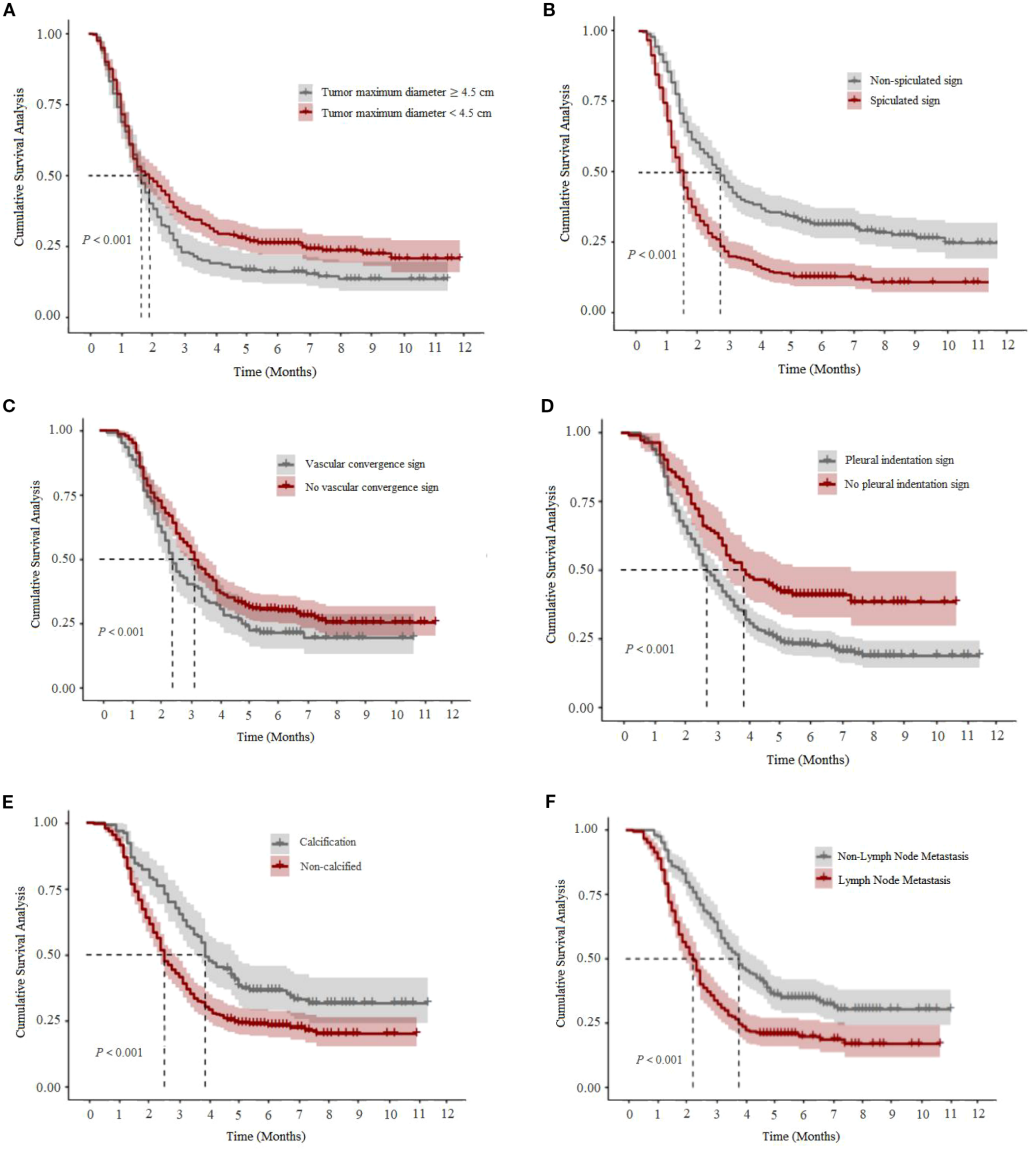
Cumulative survival analysis curve of each independent prognostic factor of the study subjects. **(A)** shows the maximum diameter of the tumor, **(B)** shows the spicule sign, **(C)** shows the blood vessel clustering sign, **(D)** shows the pleural depression sign, **(E)** shows calcification, and **(F)** shows lymph node metastasis.

COX univariate analysis showed that a total of 11 factors, including smoking history, TNM stage III-IV, tumor maximum diameter ≥4.5cm, spiculation sign, blood vessel bundle sign, pleural dent sign, chest wall invasion, pleural effusion, calcification, intrapulmonary metastasis, and lymph node metastasis, were statistically associated with the short-term prognosis of the patient (P<0.05). COX multivariate analysis showed that the maximum diameter of the tumor, spiculation sign, blood vessel clustering sign, pleural indentation sign, calcification and lymph node metastasis were independent predictive factors affecting short-term prognosis (P<0.05), see **Table 2**. The cumulative survival analysis curve of each independent factor is shown in **Figure 2**.

**Table 2:** Univariate and multivariate COX regression analysis of factors influencing short-term prognosis in NSCLC patients.

Variable	Univariate			Multivariate		
	HR	95%CI	*P*	HR	95%CI	*P*
Age (≥60 years *vs <*60 years)	1.125	0.876~1.443	0.357	–	–	–
Gender (Male *vs* Female)	1.082	0.815~1.441	0.586	–	–	–
Smoking History (Yes *vs* No)	1.326	1.015~1.734	0.038	–	–	–
Hypertension History (Yes *vs* No)	1.057	0.812~1.378	0.683	–	–	–
Diabetes History (Yes *vs* No)	1.218	0.923~1.605	0.165	–	–	–
Histological Type (Adenocarcinoma *vs* Squamous Cell Carcinoma)	1.173	0.896~1.536	0.254	–	–	–
TNM Stage (III–IV *vs* I–II)	2.315	1.782~3.007	<0.001	–	–	–
Maximum Tumor Diameter(≥4.5cm *vs <*4.5cm)	1.523	1.185~1.958	0.001	1.452	1.125~1.873	0.005
Tumor Location (Central *vs* Peripheral)	1.094	0.846~1.415	0.478	–	–	–
Well-Defined Margin (Yes *vs* No)	0.876	0.665~1.154	0.342	–	–	–
Lobulation Sign (Yes *vs* No)	1.287	0.865~1.912	0.215	–	–	–
Spiculation Sign (Yes *vs* No)	2.015	1.436~2.823	<0.001	1.893	1.324~2.705	0.001
Cavitation Sign (Yes *vs* No)	1.136	0.882~1.465	0.331	–	–	–
Vascular Convergence Sign (Yes *vs* No)	1.604	1.148~2.243	0.006	1.522	1.085~2.144	0.016
Air Bronchogram Sign (Yes *vs* No)	0.927	0.713~1.205	0.573	–	–	–
Bronchial Cutoff Sign (Yes *vs* No)	1.215	0.906~1.628	0.198	–	–	–
Pleural Indentation Sign (Yes *vs* No)	1.652	1.231~2.220	0.001	1.583	1.157~2.176	0.005
Chest Wall Invasion (Yes *vs* No)	1.375	1.056~1.792	0.018	–	–	–
Pleural Traction (Yes *vs* No)	1.186	0.908~1.552	0.207	–	–	–
Pleural Effusion (Yes *vs* No)	1.423	1.098~1.845	0.008	–	–	–
Satellite Nodules (Yes *vs* No)	1.205	0.921~1.578	0.176	–	–	–
Calcification (Yes *vs* No)	1.703	1.259~2.308	<0.001	1.622	1.195~2.204	0.002
Intrapulmonary Metastasis (Yes *vs* No)	1.825	1.386~2.407	<0.001	–	–	–
Lymph Node Metastasis (Yes *vs* No)	2.983	2.078~4.281	<0.001	2.853	1.904~4.278	<0.001

*indicates P < 0.05.

### 3.4 ROC curve

**Figure 3:**
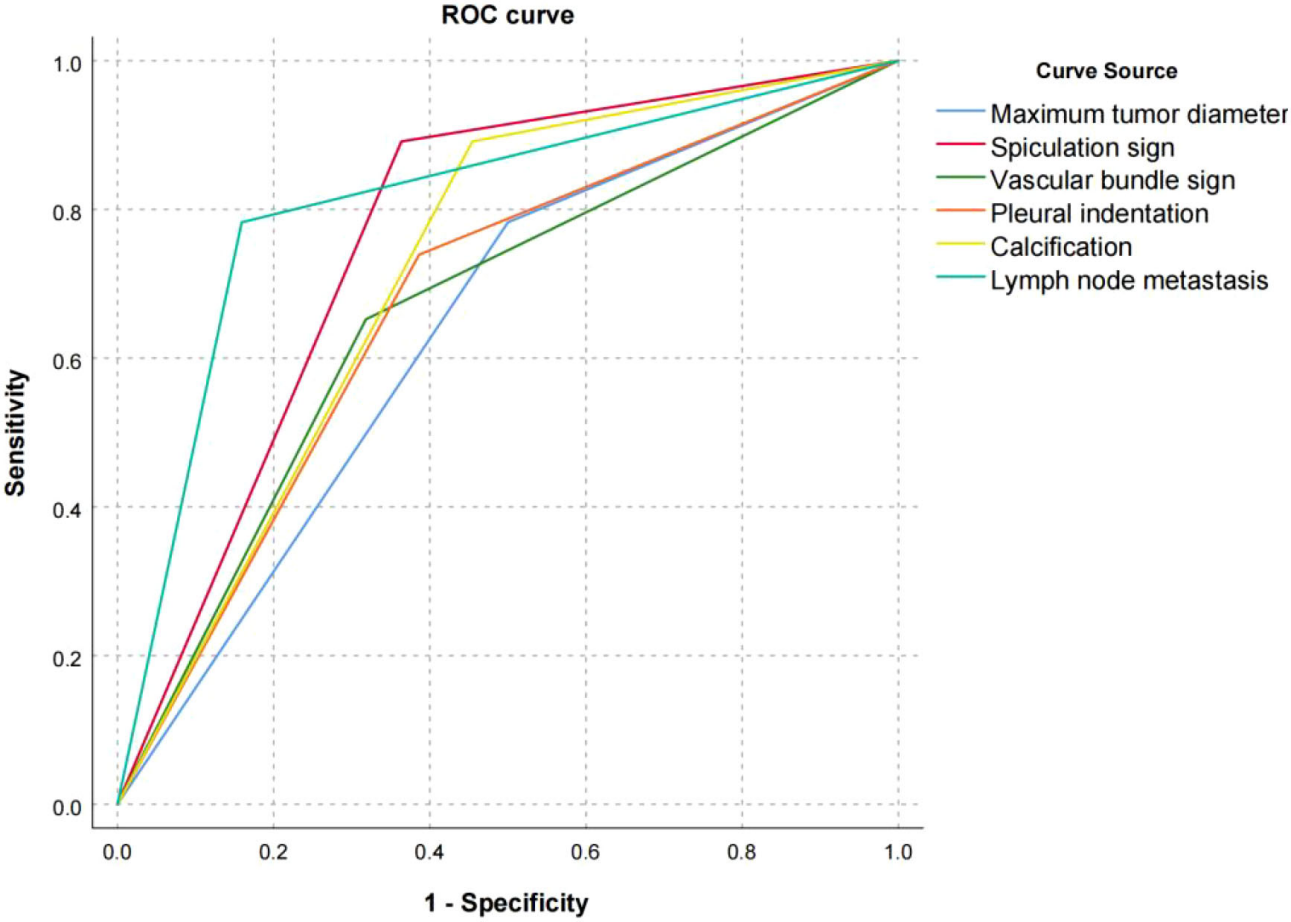
ROC curves with tumor maximum diameter, spiculation sign, vascular bundle sign, pleural indentation, calcification and lymph node metastasis, respectively.

This study used the ROC curve to evaluate the predictive value of factors such as the maximum tumor diameter, spiculation sign, vascular bundle sign, pleural indentation sign, calcification and lymph node metastasis on the short-term prognosis of NSCLC patients. The ROC curve results showed that the AUC of the maximum diameter of the tumor was 0.676 (95% CI=0.576~0.740, P=0.004), the sensitivity was 68.52%, and the specificity was 73.68%; the AUC of the spiculation sign was 0.768 (95% CI=0.663~0.847, P<0.001), the sensitivity was 68.76%, and the specificity was 72.65%; the AUC of vascular bundle sign was 0.689 (95%CI=0.610~0.763, P=0.001), the sensitivity was 67.43%, and the specificity was 72.94%; chest The AUC of membrane dent sign was 0.696 (95%CI=0.590~0.781, P<0.001), the sensitivity was 69.26%, and the specificity was 74.38%; the AUC of calcification was 0.713 (95%CI=0.614~0.808, P<0.001), the sensitivity was 69.12%, and the specificity was 73.54%; the AUC of lymph node metastasis was 0.810 (95% CI=0.716~0.886, P<0.001), the sensitivity was 78.26%, and the specificity was 81.09%; all have high predictive value, see **Figure 3**.

### 3.5 Precision-recall curve of each indicator in predicting short-term prognosis of NSCLC patients

**Figure 4:**
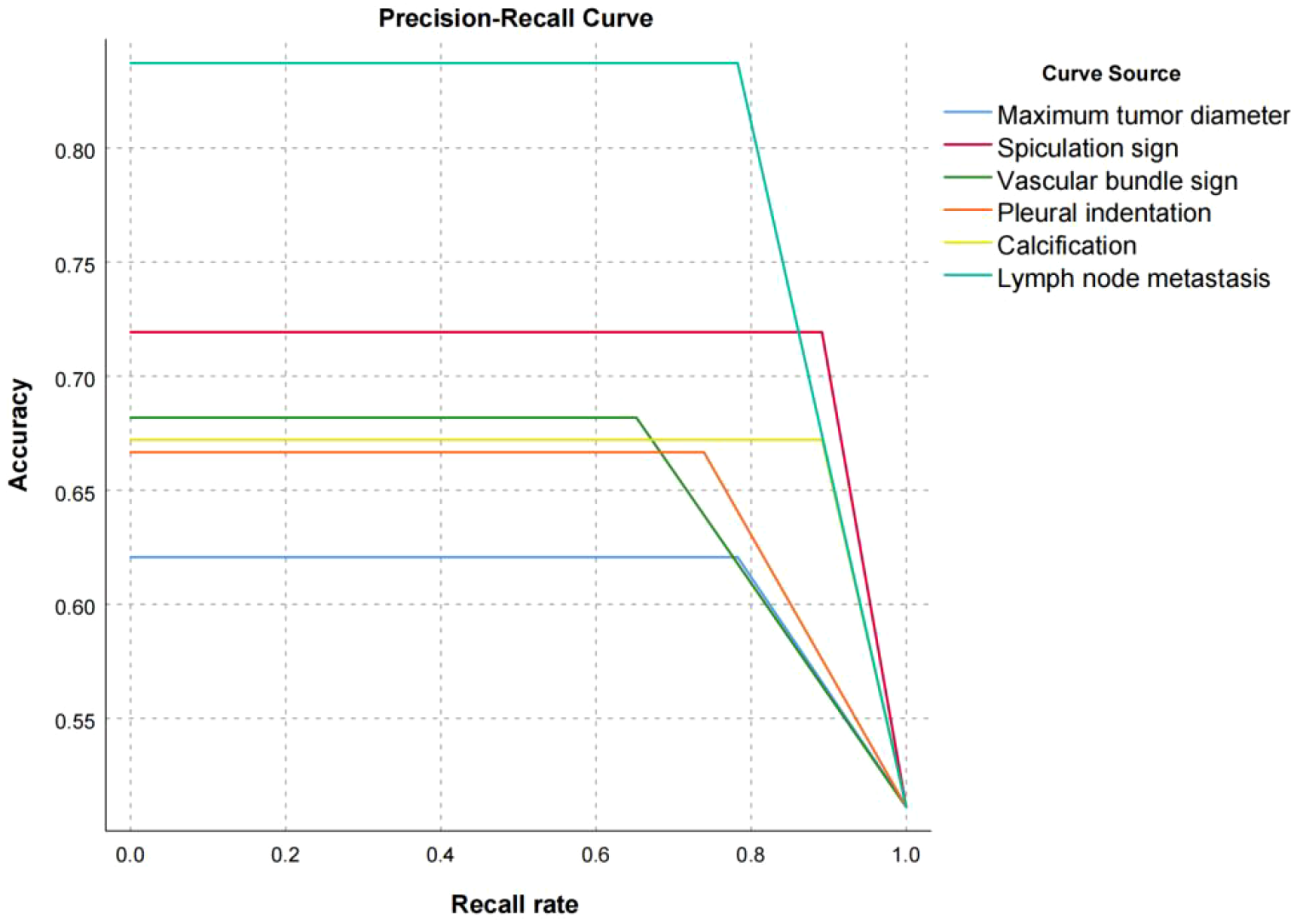
Precision-recall curves of various indicators for predicting short-term prognosis in NSCLC patients.

**Figure 5:**
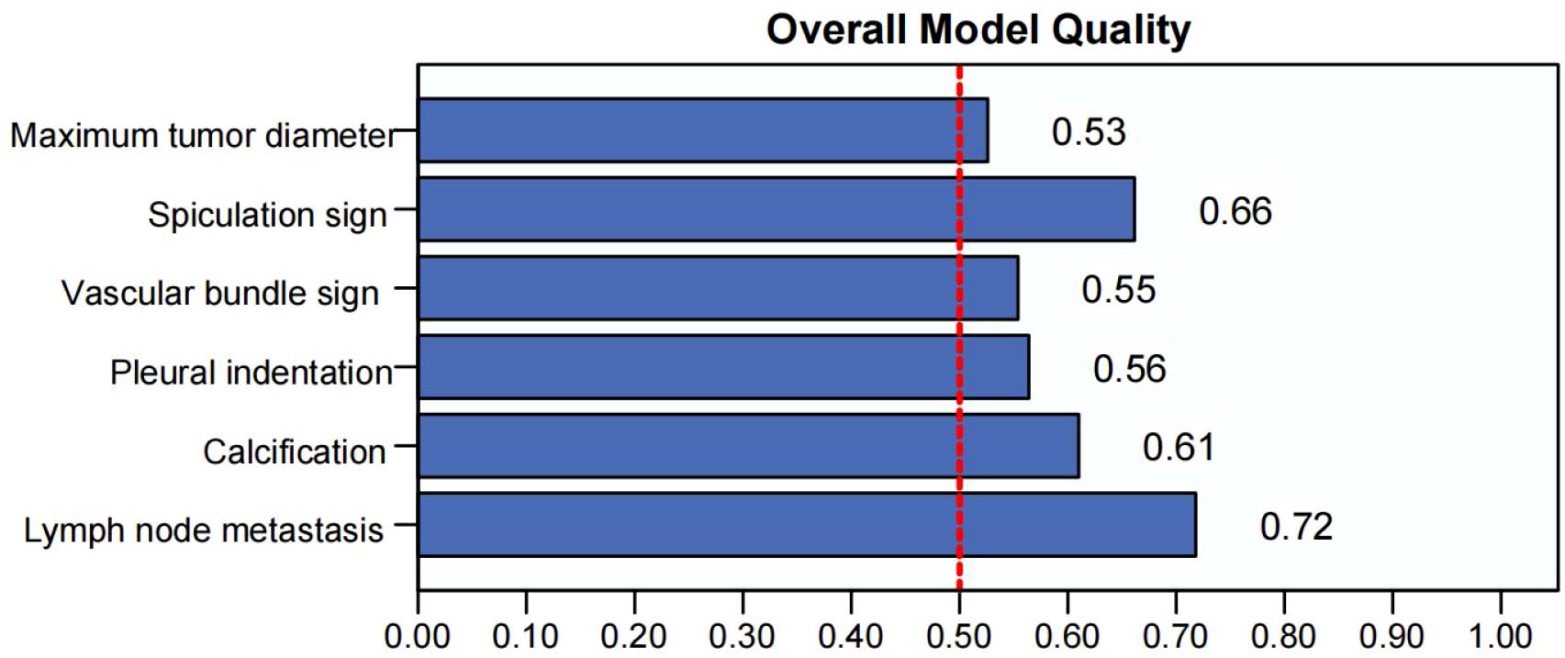
Model quality of each indicator for predicting short-term prognosis of NSCLC patients.

In the evaluation of the ability of various imaging indicators to predict the short-term prognosis of NSCLC patients, the precision-recall curve of lymph node metastasis and spiculation sign performed best. Even under high recall rates, the precision rate remained above 0.7, indicating that its prediction accuracy and stability are strong (see **Figure 4**). The model quality score is calculated based on the area under the precision-recall curve (PR-AUC). The results show that lymph node metastasis has the highest score (0.74), the spiculation sign is 0.66, and the performance is good. The other indicators are all higher than 0.5. 0.5 is selected as the cutoff threshold because 0.5 is the theoretical PR-AUC value of random prediction. If it is higher than 0.5, it means that the model prediction performance is better than the random level. Therefore, the other indicators have certain prediction capabilities, and the overall prediction effect is better than the random model (see **Figure 5**).

## 4 Discussions

NSCLC is the most common type of lung cancer with significant heterogeneity (16). The clinical manifestations are mainly respiratory symptoms such as cough, blood in sputum, and chest pain. In the middle and late stages, systemic symptoms such as fatigue and weight loss may occur (17). Because the disease progresses rapidly, early and accurate assessment and intervention directly affect patient prognosis (18). Cellina et al. (19) found that AI image analysis has significant potential in early detection of lung cancer and personalized treatment planning. The computer-assisted pulmonary nodule detection system can improve the accuracy of early detection. Chiu et al. (20) showed that AI can serve as a “second reader” in low-dose CT and chest X-ray reading, which can reduce the burden on radiologists and improve the accuracy of pulmonary nodule detection.

Based on deep learning and big data, AI image analysis software can automatically process CT, MRI, PET-CT and other imaging data of NSCLC patients, and comprehensively and accurately analyze image features. Its core advantage is precise automated processing capabilities (21–23). With the help of the AI ​​deep learning model, the software can accurately identify and segment tumor areas, extract key features such as tumor size, shape, and density, and implement standardized quantitative analysis to help doctors efficiently obtain objective data (24). It can also automatically remove artifacts caused by breathing or metal implants, improve image contrast to clearly display the lesion boundary (25), and its efficient automated processing can save doctors time in image interpretation, while achieving timely dynamic monitoring and providing support for accurate diagnosis (26). Torrente et al. (27) confirmed that AI can identify risk factors for poor prognosis in cancer patients and analyze patient characteristics, which is expected to be used in clinical monitoring and management. Kudo et al. (28) evaluated the postoperative recurrence risk of lung cancer patients based on AI analysis of three-dimensional imaging data and found that high SUVmax, larger solid tumor volume, and abnormal carcinoembryonic antigen levels were unfavorable prognostic factors that significantly affected recurrence survival rate. By analyzing image features and combining big data learning, AI software can keenly capture potential lesion patterns, assess the risk of recurrence or metastasis, and provide personalized treatment suggestions for high-risk patients (29). It not only improves the quality of NSCLC diagnosis and treatment, but also promotes the development of personalized medicine, helps doctors formulate comprehensive and accurate treatment and follow-up plans, and improves patient prognosis and quality of life.

Multivariate COX proportional hazards regression analysis in this study showed that the maximum tumor diameter, spiculation sign, blood vessel clustering sign, pleural indentation sign, calcification and lymph node metastasis are all independent factors affecting the short-term survival prognosis (progression/death within 12 months) of NSCLC patients. CT imaging characteristics (P<0.05); ROC curve analysis showed that the AUCs of the six factors were 0.676, 0.768, 0.689, 0.696, 0.713, and 0.810 respectively, all of which had high predictive value. Xie et al. (30) pointed out that the maximum tumor diameter in NSCLC patients is closely related to the risk of disease progression, recurrence and metastasis, and can predict the treatment effect and long-term prognosis: a larger tumor diameter means an increase in the number of cells and an increase in load, which suppresses host resistance by secreting immunosuppressive factors and recruiting suppressor cells. Tumor immunity also promotes angiogenesis; cell heterogeneity increases the difficulty of treatment, and drug-resistant cells are prone to remain and cause recurrence and metastasis. The hypoxic microenvironment and metabolic reprogramming (such as Warburg effect) in the tumor center will also enhance tumor invasion and proliferation ability. Therefore, the larger the tumor diameter, the higher the risk of patient prognosis. Zhang et al. (31) found that combining the CT image spiculation sign with texture features, tumor volume and other radiomic features can more accurately predict disease progression and survival: the spiculation sign reflects the highly invasive tumor growth, neovascularization and other characteristics, and its edge indicates that the tumor invades the surrounding lung tissue. The accompanying interstitial reaction and new blood vessels accelerate tumor growth. The hypoxic microenvironment and growth heterogeneity will also increase tumor permeability and recurrence risk. Li et al. (32) showed that the vascular bundle sign is closely related to the tumor proliferation rate and invasion ability. Tumors with sufficient blood supply have stronger growth and diffusion capabilities. This sign can be used as an important indicator to judge the malignancy of tumors, predict survival rate and treatment response: the vascular bundle sign indicates that the tumor invades and transforms the surrounding vascular structure and promotes the formation of new blood vessels to provide blood supply. It also indicates an enhanced hematological diffusion ability and an increased risk of distant metastasis. It also indicates that the fibrosis and hypoxic environment around the tumor aggravate the invasiveness. Chen et al. (33) found that the pleural indentation sign indicates that the tumor has a strong interaction with the pleura and surrounding tissues and has invaded the pleura or its surrounding structures. This behavior is closely related to poor short-term prognosis: the pleural indentation sign reflects the aggressive growth of the tumor and manifests as pulling the sunken pleura, indicating that the tumor breaks through the boundary of the lung parenchyma and destroys the pleural barrier to increase the risk of spread. It also indicates that the tumor is actively growing and has a high degree of malignancy. Song et al. (34) pointed out that calcification in NSCLC is related to tumor heterogeneity. Nodular or annular calcification may increase tumor invasion and metastasis potential, affecting long-term survival rate: calcification may be caused by calcium deposition in tumor necrosis or matrix remodeling and mineralization. Its presence is accompanied by increased cell heterogeneity, some cells are resistant to treatment, and may also limit drug penetration and reduce efficacy, suggesting a worse prognosis for patients. Sato et al. (35) showed that lymph node metastasis is an important sign of tumor malignancy, reflecting the ability of tumor invasion and spread, and is closely related to the low survival rate and high risk of recurrence of NSCLC patients: lymph node metastasis is an early manifestation of tumor spread. Cancer cells break through local barriers and enter the lymphatic system, and have the ability to metastasize throughout the body, indicating that tumors are more aggressive to local and distant tissues. Lymph node metastasis not only displays biological characteristics of tumor invasiveness and metastasis on CT images, but also directly affects the survival rate and prognosis of NSCLC patients.

The core contribution of this study is to establish a collaborative analysis mechanism of “artificial intelligence software initial screening + radiologist review” to achieve efficient, objective and standardized extraction of imaging features of NSCLC patients. The study not only identified multiple imaging features that are significantly related to the short-term prognosis of NSCLC, but also constructed a short-term prognosis discrimination model based on multi-factor logistic regression and ROC curve. This method has good operability and generalizability, provides a new path for the intelligentization and standardization of the lung cancer imaging assessment process, and also provides a quantitative basis for clinical precision treatment and risk stratification management.

In summary, AI image analysis software is of great value in evaluating CT imaging features that affect the short-term prognosis of NSCLC patients. It can quickly and accurately identify and quantify important risk indicators such as the maximum diameter of the tumor and spiculation sign. Through deep learning and data integration, the software automatically extracts potential patterns from imaging and clinical data, provides high-precision individualized short-term prognosis assessment, and helps identify high-risk patients and optimize treatment decisions. In addition, the software can detect subtle changes in lesions during dynamic monitoring, providing doctors with real-time risk assessment, helping to formulate more targeted treatment and follow-up plans, thereby improving the short-term prognosis of patients.

## Data Availability

The original contributions presented in the study are included in the article/supplementary material. Further inquiries can be directed to the corresponding author.
